# Nautilus at Risk – Estimating Population Size and Demography of *Nautilus pompilius*

**DOI:** 10.1371/journal.pone.0016716

**Published:** 2011-02-10

**Authors:** Andrew Dunstan, Corey J. A. Bradshaw, Justin Marshall

**Affiliations:** 1 School of Biomedical Science, University of Queensland, Brisbane, Queensland, Australia; 2 The Environment Institute and School of Earth and Environmental Sciences, The University of Adelaide, Adelaide, South Australia, Australia; 3 South Australian Research and Development Institute, Henley Beach, South Australia, Australia; 4 Queensland Brain Institute, University of Queensland, Brisbane, Queensland, Australia; University of Aberdeen, United Kingdom

## Abstract

The low fecundity, late maturity, long gestation and long life span of *Nautilus* suggest that this species is vulnerable to over-exploitation. Demand from the ornamental shell trade has contributed to their rapid decline in localized populations. More data from wild populations are needed to design management plans which ensure *Nautilus* persistence. We used a variety of techniques including capture-mark-recapture, baited remote underwater video systems, ultrasonic telemetry and remotely operated vehicles to estimate population size, growth rates, distribution and demographic characteristics of an unexploited *Nautilus pompilius* population at Osprey Reef (Coral Sea, Australia). We estimated a small and dispersed population of between 844 and 4467 individuals (14.6–77.4 km^−2^) dominated by males (83∶17 male∶female) and comprised of few juveniles (<10%).These results provide the first Nautilid population and density estimates which are essential elements for long-term management of populations via sustainable catch models. Results from baited remote underwater video systems provide confidence for their more widespread use to assess efficiently the size and density of exploited and unexploited *Nautilus* populations worldwide.

## Introduction

Nautilids have existed for over 500 million years [1], [2], [3] in a diverse and worldwide distribution. Now only two genera, *Nautilus and Allonautilus* [4] represent the extant nautiloids in a distribution restricted to deep-water tropical habitats of the Indo-Pacific. Previous studies have investigated nautiloids both in the wild and in captivity from a wide range of known populations including those in Palau, Philippines, Papua New Guinea, New Caledonia, Fiji and Australia to provide data on growth rate [5], [6], [7], [8], [9], [10], time to maturity [7], [9], [11], fertility [12], [13], [14] and lifespan [8], [11], [15], [16].

Nautiloids are traded internationally as live animals, for aquarium and possibly the pet trade; whole shells, shell worked and unworked; products for the curio, tourist, jewellery and clothing industry; and meat for exotic food markets. Most reported trade comes from the Philippines fishery but trade is also emanating from Indonesia and New Caledonia [17], [18], [19], [20], [21]. Recent studies on the Philippines fishery has shown declines in catch per unit effort of around 80% in 10–20 years, with relatively low effort by 3–4 local fishermen in each locality [19].

The life history traits of late maturity (12–15 years) [5], [9], long gestation (10–12 months) [13], [14], and long life span (20+ years) [5], [7] combined with their low fecundity (10–20 eggs per year) [13], [14] makes nautiloids particularly vulnerable to over-exploitation. The world-wide distribution of nautiloids is restricted to the Indo-Pacific region on steep coral reef drop-offs and in depths from 0–700 m [22], [23]. Nautiloids are benthic creatures which have no larvae to allow dispersal across deep ocean expanses and are limited in depth by shell implosion at around 800 m [24], [25], [26]. This limitation results in morphologically and genetically distinct populations between regions where ocean depths >800 m preclude connectivity [20]. The conservation outcome of such a distribution is that each population is subject to risk of over-exploitation with no subsequent chance of recolonization from external populations [27].

**Figure 1:**
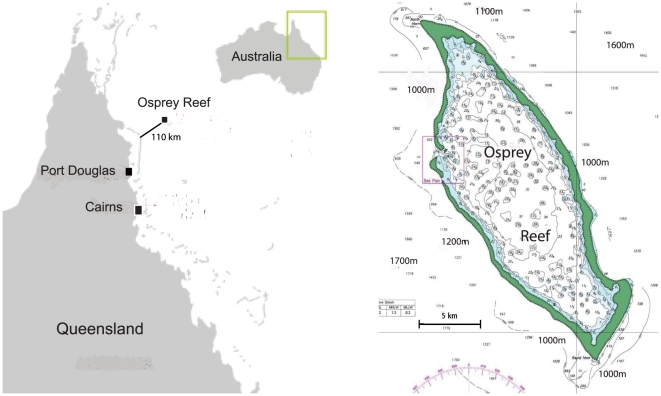
Location map of Osprey Reef, Coral Sea, Australia. Osprey Reef is located 100 km from the outer edge of the northern Great Barrier Reef, rising from around 2000 m depths to just below the surface in an almost vertical reef wall.

Osprey Reef is an isolated coral seamount surrounded by 2000 m depths with over 100 km to the next *Nautilus* population (Fig. 1). Preliminary trapping and analysis of specimens from the Great Barrier Reef and Coral Sea reefs show genetic [28] and morphological differences between each population (unpublished data). The small size (perimeter = 69.5 km) of Osprey Reef makes it an ideal site to study *Nautilus* demography and behaviour.

While Osprey Reef is a closed system to *Nautilus*, with no export of individuals and no recruitment from other sources, we still classify it as an open population due to the long period of our study and the sometimes rapid birth and death rates observed. We used mark-recapture to estimate population size and additional data collected from automated underwater vehicles, baited remote underwater video systems and standard trapping to describe home range size and estimate densities. Population estimates are essential elements in the analysis of the conservation status of these genera under the International Union for the Conservation of Nature (IUCN) Red List Criteria (www.iucnredlist.org) and under the Convention on the International Trade of Endangered Species (CITES) listing criteria for protecting species that are in international trade (www.cites.org). Ours is the first study to provide estimates of population size and density as a preliminary baseline for such assessments.

## Results

### Capture, mark and recapture

**Figure 2:**
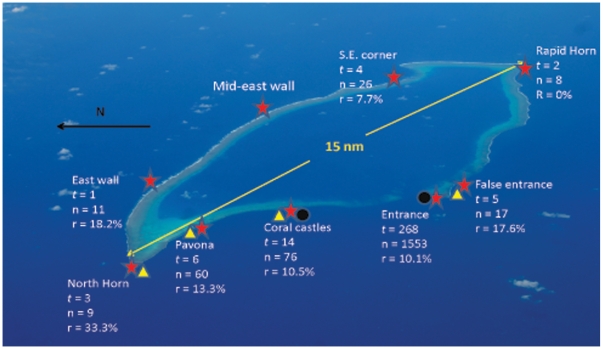
Osprey Reef: *Nautilus* sampling and tracking detection sites. *Nautilus* trapping was done at eight sites (red stars, *t* = number of samples at each site around Osprey Reef totalling 303 samples of which 92% were at the Entrance site, *n* = total number of *Nautilus* captured and r = % recaptures of total capture number). Baited Remote Underwater Video System deployments (black circles) were undertaken at two sites while Remotely Operated Vehicle dives (yellow triangles) were done at four sites with two dives at both Pavona and Coral castles. A VR2 receiver at Mid-East Wall recorded the presence of one *Nautilus* released at Entrance.

We deployed and successfully retrieved 268 traps at the Entrance site, Osprey Reef, from August, 2000 to January, 2006, resulting in the capture of 1553 individual *Nautilus* (1203 males, 251 females and 99 immature/juveniles) and the recapture of 157 individuals (Fig. 2). *Nautilus pompilius* was the only species captured. There was some indication of a seasonal trend for sampling maxima towards the middle months of the year; however, catches within the 95% confidence interval of mean catch rates (6.4 trap^−1^) were returned throughout all months, with February being the only month without sampling effort (Fig. S1).

Trapping data show a male∶female sex ratio of 83∶17 from animals mature enough to determine sex and an age distribution of 0.002% juvenile *Nautilus*, 42% sub-adult and 58% mature individuals (mature animals were identified by the presence of a black apertural margin; in juveniles color banding is continuous to aperture and shell length is <90 mm). Trapping with smaller mesh traps at depths ranging from 100 to 450 m showed no change in the sex ratio or age frequency. The smallest *Nautilus* captured had a shell diameter of 76 mm; we could not reliably determine sex for individuals <115 mm diameter (i.e., immature or juvenile). We recaptured 9.5% of marked individuals; all recaptures were individuals that had been initially captured and released at the Entrance site.

### Model ranking

Analysis of capture matrices (mature/sub-mature and immature individuals) without the sex parameter by the Cormack-Jolly-Seber bootstrap goodness-of-fit method, identified the time-dependant model 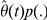 as having the highest support (assessed via Akaike's information criterion corrected for small samples – AIC*_c_*). The χ^2^goodness-of-fit analysis showed the model fit the data well (χ^2^ = 0.992; P = 0.55) with no evidence for over-dispersion (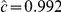). Analysis of capture matrices (mature/sub-mature individuals) with the sex parameter by the Cormack-Jolly-Seber method identified the model with time (*t*) and sex (*s*) effects on both the probabilities of survival (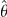) and capture (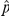),
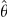
(s*t)*p*(s*t)β(.)*N*(.), as having the highest bias-corrected model support (Table 1). These are presented in full in Tables S1& S2.

**Table 1:** Bootstrap Goodness goodness-of-fit summaries for population models.

Sex	Model	*w*AIC*_c_*	*k*	*P*	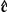
N	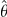 (*t*)*p*(.)	0.84561	19	0.55	0.992
N	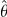 (*t*)*p*(*t*)	0.15439	39	0.44	1.031
Y	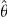 (s*t)*p*(s*t)	0.38933	43	0.18	1.142
Y	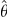 (*t*)*p*(.)	0.00046	16	0.34	1.040

Two different matrices were analysed; one with all individuals (1553 total) present and one with only individuals having sex determined (1360 total) (sex parameter: Y/N column). Models shown are the highest ranking models and include probability of survival (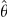) and capture (*p*) with combinations of sex (*s*) and time (*t*). Shown are Akaike's information criterion corrected for small samples (AIC*_c_*), quasi-AIC*_c_* weights (*w*AIC*_c_*), the number of estimable parameters (*k*), probability of a deviance less than or equal to the observed deviance from 100 bootstrap goodness-of-fit simulations of the model (*P*), and the estimated quasi-likelihood over- (or under-) dispersion factor (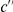).

Model-weighted averages for apparent survival parameters for total individuals, males and females were 0.733±0.086, 0.883±0.389 and 0.761±0.132, respectively. Model weighted averages for recapture parameters for total individuals, males and females were 0.010±0.010, 0.0138±0.0053 and 0.017±0.006, respectively.

### Population estimate

Using the POPAN open-population Jolly-Seber model structure implemented in MARK, the data without the sex-dependent parameter gave a top-ranked time-dependent model 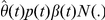. Similar analysis with the sex parameter gave a top-ranked time-dependent model 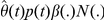,without time dependency of the probability of entry (*β*) parameter (Table S3). POPAN-derived estimates of population size were compared for the top-ranking models with and without the sex parameter. Mean total, male and female temporal population estimates varied by maximums of 12.2, 16.6 and 34.7% respectively between models (Table 2).

**Table 2:** POPAN population estimate results.

Total population estimate		*N*		95% CI		
Sex	Model	Mean	SD	Low	High	CV
Y	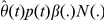	2344	625.9	1117	3570	26.7
Y	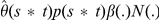	2404	621.8	1185	3623	25.9
Y	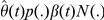	2731	722.0	1316	4146	26.4
N	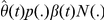	2688	751.5	1215	4161	30.1
N	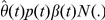	2497	677.3	1169	3824	27.1
N	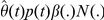	2656	924.2	844	4467	34.8

Population estimates from the two different matrices were analysed; one with all individuals (1553 total) present and one with only individuals having sex determined (1360 total) (sex parameter Y/N column). Models shown are the highest-ranking models and include probability of survival (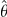) and capture (*p*) with combinations of sex (*s*) and time (*t*). Shown are population estimate (*N*) mean and standard deviation (SD) with low and high 95% confidence intervals bounds (95% CI) and coefficient of variation (CV). Estimates for total population from both matrices and for males and females from the sex parameter models are shown.

From the top-ranked model without the sex parameter, 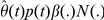 we estimate 2656 individual adult/sub-adult and immature *Nautilus* (95% CI = 844 to 4467; SD = 924). The same model with the sex parameter and including only mature/sub-mature individuals estimates 2344 individual mature/sub-mature *Nautilus* (95% CI = 1117 to 3570; SD = 626), comprising means of 1900 males (95% CI = 906 to 2896; SD = 509) and 443 females (95% CI = 211 to 675; SD = 118).

**Figure 3:**
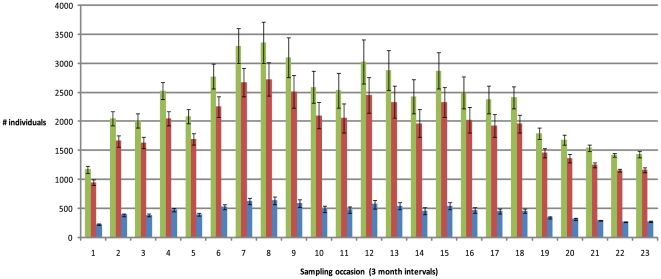
Temporal population estimates for total individuals and total males and females (mature/sub-mature individuals). Estimates of total population (black bars), total males (grey bars) and total females (light bars) for each pooled three-month sampling occasion from the time-dependent model from the capture matrix with sex distinction, 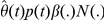. Error bars (1 standard error of the mean) are shown. Mean population estimates over the entire sampling period are 2344 individual mature/sub-mature *Nautilus* (95% CI = 1117 to 3570; SD = 626); 1900 males (95% CI = 906 to 2896; SD = 508) and 443 females (95% CI = 211 to 675; SD = 118).

**Figure 4:**
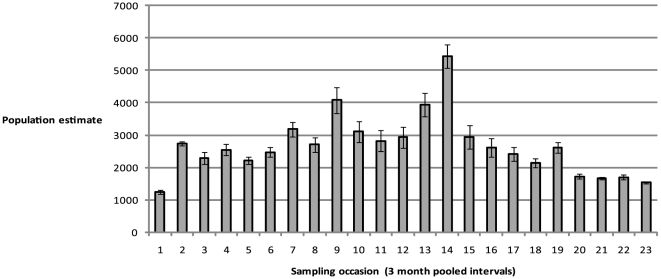
Temporal population estimates for total individuals (mature/sub-mature and immature individuals). Estimates of total population for each pooled three-month sampling occasion from the time-dependent model from the capture matrix for all individuals, without sex distinction, 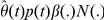.Error bars (1 standard error of the mean) are shown. Mean population estimate over the entire sampling period is 2656 individual adult/sub-adult and immature *Nautilus* (95% CI = 844 to 4467; SD = 924).

There were temporal trends in population size estimates (Fig. 3 & 4). The sex parameter model with only mature and sub-mature individuals ranged between 1172 and 3363 total individuals, while the model without the sex parameter and including immature animals ranged between 1237 and 5441 individuals.

### Effects of sex, tag retention and tag-induced mortality

We investigated the effects of sex on population estimates by analysing all combinations of models including the effects of sex (*s*) and time(*t*) on the probabilities of survival (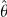) and capture (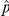).While the sex linked parameter models were highest ranked in Cormack-Jolly-Seber goodness-of-fit tests, this was not the case in the POPAN analysis. Population estimates were only subtly different (12.2% and well within 95% CI bounds) between the various models and indicate that any sex effect was minimal.

Tagged immature to mature *Nautilus pompilius* are expected to live for between 0 to 12+ years from capture time, by interpolating growth and age estimates [5] if no tag-induced mortality occurs. We assessed tag-induced mortality by model-weighted average apparent survival estimates (total individuals, males and females were 0.733±0.086, 0.883±0.389 and 0.761±0.132, respectively) which are within expectations for minimal tag mortality effect.

We estimated tag retention by comparing digital images of tagged animals from initial capture to recapture up to 5+ years later. We also compared digital shell fingerprint images providing unique individual identification for all animals captured to ensure no tags were missed or misidentified. All tagged animals were correctly identified on recapture and no tag loss was evident from image or live comparison.

### Baited remote underwater video system attraction times

**Figure 5:**
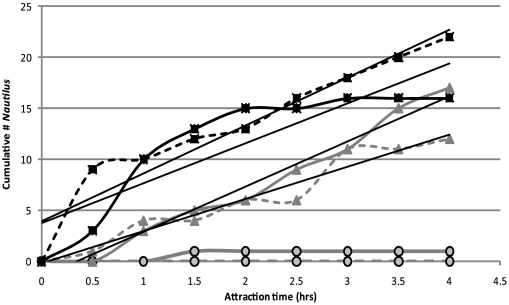
Baited Remote Underwater Video System *Nautilus* attraction times. The cumulative number of *Nautilus* attracted to the baited trap over time is shown to produce a linear curve with a mean attraction rate of 4.03 hr^−1 ^for the red (grey triangles) and blue/white/red (black squares) lighted traps. Traps with blue light only (black outlined circles), showed almost no attraction. Replicated results for each light combination are shown.

Analysis of video data from video system deployments showed a negative effect on attraction when blue lights were used. These data were subsequently excluded from density and attraction analysis. Results of red light and a combination of red, white and blue light systems deployed within 200 m of each other were similar. There was a linear increase in cumulative *Nautilus* numbers attracted to the video system over time, with a mean attraction rate of 4.03 hr^−1^ (Fig. 5). From video observations we could not distinguish all individuals to sex or maturity status. We did identify only 3 juveniles out of a total of 68 *Nautilus* recorded.

### Remotely operated vehicle daytime sighting frequencies and depth distribution

Remotely operated vehicle data from video footage of daylight dives (reported in more detail in a concurrent publication [22]) showed a maximum encounter rate in the 500–700 m depth range. *Nautiluses* were recorded from 450 to 703 m; however, none were sighted between 100 and 450 m. All individuals observed were in foraging mode, swimming actively and with feeding tentacles extended and there was an attraction to the remote vehicle, and specifically towards the white lights in many of the observed animals.

There was a mean sighting frequency of 11.3 km^−1^ of reef for the four sites on Osprey Reef where dives covered the overall *Nautilus* vertical distribution zone from 100–700 m (Table 3). Osprey Reef has a perimeter of suitable steep reef wall *Nautilus* habitat of 69.5 km in total. Using the average slope of 50°from bathymetric data [29] results in a calculation of 57.7 km^−2^ of *Nautilus* habitat, a density estimate of 13.6 km^−2^ and total Osprey Reef estimate of 785 individuals active during daytime at depths between 100–700 m.

**Table 3:** Remotely operated vehicle observations of *Nautilus* during daylight dives.

Location	# *Nautilus*	Horizontal survey distance	individuals km^−1^
Coral Castles	13	1.195	10.9
North Horn	10	0.790	12.7
Pavona	15	1.451	10.3
False Entrance	10	0.806	12.4
**Total**	**48**	**4.242**	**11.3**

Daylight dives at four sites on Osprey Reef show total number of *Nautilus* recorded (# *Nautilus*), the horizontal survey distance and calculation of the number of individuals km^−1^.

### Movement and home range

Speed of movement calculated from the baited video analysis provided a mean maximum speed of 0.82 km hr^−1^. Longer-term vertical movements obtained from telemetry studies show vertical migrations of 300–400 m at a mean velocity 0.18 km hr^−1^. Short-term horizontal tracking detections showed maximum speeds of 1.18 to 0.61 km hr^−1^for periods from 110 to 317 minutes exhibited by three males. Horizontal tracking resulted in maximum recorded longer-term movements from 5.5 to 29.3 km over 9 to 52.3 days with a mean movement rate of 1 km day^−1^. (Table S4).

Percentage recapture from all Osprey Reef sampling sites (Table S5) show consistent recapture rates around the reef perimeter from sites with >5 samples. All sites, with the exception of Rapid Horn, recorded recaptures. Current strength below 150 m was negligible as determined from baited video and remote vehicle footage, observations by the experienced operators, and the drift of fine sediment particles. The formation of the reef slope also indicates low current as there are vertical ‘sediment rain’ grooves and a lack of filter-feeding organisms below 150 m but the presence of suspension feeding crinoids and echiurid worms.

## Discussion

Population estimation is the first step in determining the conservation status of a species by providing a temporal and numerical reference point from which to assess change. For clandestine marine species this can be particularly difficult. Population estimates are challenging for *Nautilus* due to its deep ocean habitats which are beyond the depth range of observational diving, its presence in remote locations, and unknown elements of its early life. Capture-mark-recapture techniques in conjunction with the introduction of more recent technology, remote vehicles and baited deep water high definition cameras, have enabled us to estimate the population size and density of *Nautilus* at a single locality for the first time. These methods also provided further insight into the sex and age structure of wild populations.

Reports of rapid declines in catch per unit effort in the Philippines *Nautilus* fishery [19] and by inference, abundance, has highlighted the need to calculate sustainable catch rates. Population estimates and demographic data are essential components of effective management of nautiloid fishing in existing fisheries and are especially valuable elements for the management of nautiloid habitats as yet unexploited. Our population estimates of *Nautilus pompilius* at Osprey Reef assume that we effectively sampled the entire *Nautilus* habitat of this reef and could demonstrate that local sampling is representative of the entire area occupied by the species. Trapping data from sampling sites around the perimeter of Osprey Reef support these assumptions by indicating that *Nautilus* travel around the entire reef and that animals trapped at the Entrance site have a similar recapture probability to all sites (Fig. 1 & Table S5). Acoustic telemetry data reveal travel rates up to 3.2 km in less than one day, maximum speeds of up to 1.18 km hr^−1^ for short periods (<6 hours) and around 1 km day^−1^ averaged over >9 days (Table S4). Speed estimates made from video footage (0.82 km hr^−1^) and previous reported rates of 0.54 km hr^−1^ [30] are in approximate agreement. Weak currents at depths >200 m suggest that *Nautilus* movement rates arise principally from self-propulsion, giving an estimated minimum time of 70 days to navigate the perimeter of Osprey Reef. Thus, sampling at the Entrance site provides a representative sample of the Osprey Reef population when taken over at least 3 months.

The population is dominated (83%) by males, which is similar to the sex ratio reported in other *Nautilus* populations in the Philippines [31], Palau [32], New Caledonia [23] and Papua New Guinea [33]. The age structure consists of 58% adults, 42% sub-adults and only 0.002% juveniles (3 individuals). Observations from a remotely operated vehicle recorded a much higher percentage (10%) of juveniles as did records from baited video system footage (4.4%). These data also indicate that juveniles live within the same vertical distribution regime as adults [22], [34]. This information refutes the notion that *Nautilus* displays ontogenetic habitat partitioning and instead suggests that juveniles are indeed rare [3], [9], [31]. Indeed, there are few data collected elsewhere describing the juvenile component of the population beyond occasional trapping [9], [23], [31], [35], [36], [37], [38], [39] and remote camera data [34], [40]. These data indicate that juveniles represent <10% of the population). This result reconfirms that *Nautilus* has low fecundity.

The population abundance models used to analyse the data obtained in this study estimated total population size within 12.2% of each other and well within their respective 95% confidence interval limits. Male population estimates among models were similarly within a range of 16.6%, while the less-sampled females provided more variable estimates among models (within 34.7% of each other). Combined, our various estimates suggest that the total median *Nautilus* population of Osprey Reef is around 3000 individuals.

The Cormack-Jolly-Seber method assumes that (1) every marked animal present in the population at time *i* has the same probability of recapture p*_i_*, (2) every marked animal in the population immediately after time *i* has the same probability of surviving to time *i*+1, (3) all samples are instantaneous relative to the interval between occasion *i* and *i*+1, and (4) each release is made immediately after the sample [41]. Our evidence suggests that we meet these assumptions given the constraints and methodologies used to undertake the current research. The survival rate of tagged animals is high, as demonstrated from apparent survival results (0.733±0.086). Issues such as stress upon capture or during retention time were minimised by using cooled dark, aerated holding tanks and night release at 30 m, away from visual predators. Only two individuals were confirmed to have died between capture and release (due to a near surface release at night in a boat-light area and subsequent predation). No loss of any marks was observed during the study by both visual inspection and validation using digital shell fingerprint images. For the assumption of instantaneous release, we were compelled to pool individuals within three-month bins, and release was within 12 hours after each sample.

Video observations provide information that supports the general mark-recapture population estimates, albeit with a substantial downward bias. From the remote vehicle data, we estimated a density of 13.6 daytime-active individuals km^−2^ of reef in the zone from 100–700 m. This equates to 32% of the 45.8 *Nautilus* km^−2^ density estimated by capture-mark-recapture methods. Acoustic telemetry data indicate that Osprey Reef *Nautilus* tend to rest, possibly cryptically, at 200 m during the day [22] which could explain why only a relatively low percentage of *Nautilus* were observed by the ROV actively foraging during the day.

Consistent baited remote underwater video system attraction rates of 4.03 hr^−1^ suggest a relatively even distribution of Nautilus throughout the area sampled. When combined with known population numbers (2656 individuals) and known total habitable area of the population (57.7 km^−2^) these attraction rates allow calculation of the hourly area sampled by the equipment (hourly sampling area = 4.03/2656 * 57.7 km^−2^ = 0.088km^2^ hr^−1^).

Baited remote underwater video systems may provide an efficient new method to estimate *Nautilus* population density at other locations or at the very least provide a standardized comparative measure of abundance between populations [42], [43].

### Conclusions

Our data indicate that Osprey Reef is sparsely populated by *Nautilus pompilius* relative to catch reports from other populations [19]. Despite its low abundance, the species is easily attracted to baited traps and so can be taken efficiently by fishers. Indeed, small-scale fisheries can rapidly induce declines in catch rates [19]. As such, our population estimates and other measurements of the species' life history traits [5] represent essential information for estimating sustainable *Nautilus* fishing targets. Although *Nautilus* fishing is not permitted at Osprey Reef, our data and approach provide those tasked with managing other populations with the necessary tools for sustainable use. Further, with the assistance of species experts, existing and new biological data on *Nautilus* and *Allonautilus* species are currently being evaluated to determine their conservation status and the impact of international trade. The results of this study are therefore a critical step forward in this process.

## Materials and Methods

### Ethics statement

Research was conducted under permit from the Australian Fisheries Management Authority and with ethics approval from the University of Queensland Animal Ethics Committee.

### Capture-mark-recapture

Barrel-shaped traps (90×77 cm) constructed from wire mesh (7.5×9 cm mesh size) baited with chicken meat and set to 300 m depth were used to trap *Nautilus*. Sampling was done between 01/08/2000and 17/01/2006 throughout the year (with the exception of February only having one sample). The nine sample sites were located around the entire circumference of Osprey Reef in the Coral Sea of Australia (13°54.190′S, 146°38.985′E; Fig. 1) however 92% of sampling was undertaken at the Entrance site (Fig. 1). Traps were deployed for 12 hours, set at dusk and retrieved vertically at dawn at 15 mmin^−1^ with *Nautilus* immediately placed in a dark refrigerated tank to contain the animals during the tagging process at temperatures between 16 and 19° C.

We engraved the shell of captured individuals with a unique number, photographed, and measured for diameter and aperture width, and maturity (presence of black apertural margin; juveniles have banding continuous to aperture and shell length <90 mm) and sex (presence of spadix or nidimental gland). Recaptured animals were re-photographed, measured and sexed before release. Tags were always visible but identity of each recapture was checked later using the unique ‘fingerprint’ of each individual shell pattern. Immature animals at the time of first capture showed a distinct apertural marking (‘shock line’) from the capture event followed by further normal apertural growth, both of which were easily visible in recaptured specimens. All animals with this ‘shock line’ were checked for numbers which verified tag retention and identification. To reduce the chance of predation by visual predators upon release, captured individuals were kept on board for 13 hours prior to release after dark. Animals were checked for neutral buoyancy and normal swimming and descent behaviour during release on scuba at 30 m at the capture location.

### Population estimate

Full capture and recapture data from 01/08/2000–17/01/2006 at the Entrance site was entered into a capture matrix for analysis with program MARK. We constructed one matrix incorporating all capture events from the full study period as well as matrices which pooled individual capture events into one, three and six-month capture events. The three-month pooled data provided the best model fits. From this dataset we produced two matrices: one for all individuals (1553 total) and a second matrix to include an individual's sex (1360 individuals because 193 animals were too immature to determine sex conclusively). A bootstrap goodness-of-fit procedure (Cormack Jolly Seber method) was used to determine the best model to estimate population number using the POPAN method [44]. We could not estimate the size of the juvenile population due to sample size constraints, but we estimated what proportion of the entire population juveniles comprise using baited remote underwater video systems and remotely operated vehicle footage and trapping records.Within program MARK the sex parameter was explored to determine its effects on probabilities of survival or capture and tag retention, and tag-induced mortality were also examined as a source of error in population estimation. Sex contributed minimal error and therefore we disregarded it in estimates. We analysed all combinations of models including the effects of sex (*s*) and time (*t*) on the probabilities of survival (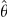) and capture (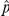).

We applied an open-population Jolly-Seber model [41] to the mark-recapture data using the POPAN option in the program MARK [44] to estimate population size. For *t* capture occasions the model provides *t*−1 estimates of *θ* (apparent survival), *t* estimates of p (capture probability given the animal is alive and available for capture), *t*−1estimates of *β* (probability of entry into the population per occasion), and *N* (super-population size). Models were fitted using the logit link function for 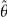 and 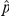, the identity link function for 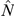, and the multinomial logitlink function to constrain the set of 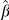 parameters to ≤1(otherwise, convergence can be problematic [44]). The number of parameters for each model was adjusted to account for parameters not estimable due to low recovery rates in certain years. We used Akaike's information criterion corrected for small sample sizes (AIC*_c_*) to compare models and provide model-averaged estimates of *N* [45]. Population estimates were obtained for the top ranking of both sex and non-sex parameter models for both Cormack-Jolly-Seber goodness-of-fit and POPAN analyses. Model-weighted averages of apparent survival and recapture parameters were developed under the full Cormack-Jolly-Seber [44] mark-capture framework.

### Baited remote underwater video systems

A baited remote underwater video system consists of a steel T-shaped frame with or without a baited trap attached at one end a distance of 30 cm from a Sony HD camera and single light source mounted on the T section. The unit can record for up to 6 hours. LED lights of red, blue or white wavelengths were used and the video systems were deployed at 300 m depth on the near-vertical reef walls of Osprey Reef at two different sites (Fig. 1). Deployment and retrieval was the same as for standard trapping methods. Video footage was reviewed and logged for *Nautilus* presence and behaviour.

### Remotely operated vehicle (ROV) observations

A Cherokee ROV capable to 800 m was deployed at Osprey Reef for six daytime observations at four different sites (Fig. 1) for dives of between four to eight hours with a total of 29.05 hours search time and 4.242 km distance. Standard mini-DV footage and still images using white halogen light sources were taken alongside real-time linked observer data recording. Records of depth and location of the vehicle were then matched with video and written records of habitat type, *Nautilus* presence, light transmission, and current strength and direction.

### Movement rates

We estimated maximum movement rate for animals of known size by observing footage of mature male animals (mean diameter 134 mm) a known distance from the camera and moving perpendicular to the camera across the entire field of view. Time to move a set number of animal lengths provided speed of movement for video segments at maximum locomotion. Three animals were analysed for a series of three maximum movement rates.

## Supporting Information

Figure S1 **Seasonality of** ***Nautilus*** **trapping at Osprey Reef.** Mean catch rate (# *Nautilus*/trap) for each month with number of samples and overall mean (6.4 *Nautilus*/trap). Error bars are 1 standard error of the mean.
(EPS)

Table S1
**Cormack-Jolly-Seber bootstrap goodness-of-fit results.** The16 top-ranked models from the capture matrix with only individuals having sex determined (1360 individuals) are shown. Models include probability of survival (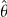) and capture (*p*) with all combinations of sex (*s*) and time (*t*). Shown are Akaike's information criterion corrected for small samples (AIC*_c_*) difference between the top-ranked model AIC*_c_* and the current model (äAIC*_c_*), AIC*_c_*weights (*w*AIC*_c_*) and the number of estimable parameters (*k*). From bootstrap goodness-of-fit tests the probability of a deviance less than or equal to the observed deviance from 100 bootstrap goodness-of-fit simulations of the model (*P*), and the quasi likelihood over (or under) dispersion factor (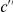) area also presented.(DOCX)

Table S2
**Cormack-Jolly-Seber bootstrap goodness-of-fit results.** All models from the capture matrix with all individuals (1553 individuals) are shown. Models include probability of survival (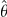) and capture (*p*) with time (*t*). Shown are Akaike's information criterion corrected for small samples (AIC*_c_*) difference between the top-ranked model AIC*_c_* and the current model äAIC*_c_*), AIC*_c_*weights (*w*AIC*_c_*) and the number of estimable parameters (*k*). From bootstrap goodness-of-fit tests the probability of a deviance less than or equal to the observed deviance from 100 bootstrap goodness-of-fit simulations of the model (p-value), and the quasi likelihood over (or under) dispersion factor (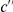) area also presented.(DOCX)

Table S3
**POPAN goodness-of-fit summaries for highest ranking population models.** Two different matrices were analysed; one with all individuals (1553 total) present and one with only individuals having sex determined (1360 total) (sex parameter Y/N column). Models shown are the highest ranking models and include probability of survival (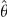) and capture (*p*) with combinations of sex (*s*) and time (*t*). Shown are Akaike's information criterion corrected for small samples (AIC*_c_*), AIC*_c_*weights (*w*AIC*_c_*) and the number of estimable parameters (*k*).(DOCX)

Table S4 ***Nautilus*** **horizontal movement speeds from remote telemetry studies.** *Nautiluses* tagged with ultrasonic transmitters were tracked for extended periods. Records of maximum speeds over short term (<6hrs) and longer term (>7 days) are shown for individual tagged animals (Nautilus ID) of known sex and shell diameter. Distance travelled and time taken is presented with the average speed calculated.
(DOCX)

Table S5 ***Nautilus*** **capture and recapture data from Osprey Reef sampling sites.** Data for *Nautilus* captures and recaptures at all sampling sites on Osprey Reef. All recaptures were originally trapped at the Entrance site and recaptures at other sites demonstrate the movement of individuals around the entire perimeter of Osprey Reef (Fig. 2).
(DOCX)
