# Supplementary material for: Nautilus at Risk – Estimating Population Size and Demography of Nautilus pompilius
Source: PLoS One. 2011 Feb 10;6(2):e16716. doi: 10.1371/journal.pone.0016716 (PMC3037370; doi:10.1371/journal.pone.0016716)
Supplement: Table S1 — Cormack-Jolly-Seber bootstrap goodness-of-fit results. The16 top-ranked models from the capture matrix with only individuals having sex determined (1360 individuals) are shown. Models include probability of survival () and capture (p) with all combinations of sex (s) and time (t). Shown are Akaike's information criterion corrected for small samples (AICc) difference between the top-ranked model AICc and the current model (äAICc), AICcweights (wAICc) and the number of estimable parameters (k). From bootstrap goodness-of-fit tests the probability of a deviance less than or equal to the observed deviance from 100 bootstrap goodness-of-fit simulations of the model (P), and the quasi likelihood over (or under) dispersion factor () area also presented. (DOCX) [file pone.0016716.s002.docx]

**Table S1.Cormack-Jolly-Seber bootstrap goodness-of-fit results.**

| Model | AICc | δAIC*_c_* | *w*AIC*_c_* | *k* | P | $\hat{c}$ |
| --- | --- | --- | --- | --- | --- | --- |
| $\hat{\theta}$(*s***t*)*p*(*s***t*) | 1267.954 | 0 | 0.38933 | 43 | 0.18 | 1.142 |
| $\hat{\theta}$(*t*)*p*(*s***t*) | 1268.706 | 0.752 | 0.26731 | 40 | 0.03 | 1.254 |
| $\hat{\theta}$(*.*)*p*(*s***t*) | 1269.23 | 1.2759 | 0.20571 | 31 | 0.10 | 1.151 |
| $\hat{\theta}$(*s*)*p*(*s***t*) | 1270.783 | 2.8295 | 0.0946 | 32 | 0.05 | 1.210 |
| $\hat{\theta}$(*.*)*p*(*s*) | 1273.831 | 5.8776 | 0.02061 | 3 | 0.14 | 1.144 |
| $\hat{\theta}$(*s*)*p*(*s*) | 1275.281 | 7.3268 | 0.00998 | 4 | 0.06 | 1.157 |
| $\hat{\theta}$(*t*)*p*(*s*) | 1275.982 | 8.0285 | 0.00703 | 18 | 0.08 | 1.195 |
| $\hat{\theta}$(*t*)*p*(*t*) | 1279.551 | 11.5974 | 0.00118 | 32 | 0.32 | 1.048 |
| $\hat{\theta}$(*s***t*)*p*(*s*) | 1279.615 | 11.6615 | 0.00114 | 25 | 0.21 | 1.079 |
| $\hat{\theta}$(*s*)*p*(*t*) | 1279.819 | 11.8648 | 0.00103 | 25 | 0.08 | 1.197 |
| $\hat{\theta}$(*s*)*p*(*,*) | 1280.24 | 12.2862 | 0.00084 | 3 | 0.10 | 1.151 |
| $\hat{\theta}$(*.*)*p*(*t*) | 1281.397 | 13.4436 | 0.00047 | 24 | 0.51 | 0.991 |
| $\hat{\theta}$(*t*)*p*(*.*) | 1281.441 | 13.4868 | 0.00046 | 16 | 0.34 | 1.040 |
| $\hat{\theta}$(*.*)*p*(*.*) | 1282.354 | 14.4005 | 0.00029 | 2 | 0.43 | 1.012 |
| $\hat{\theta}$(*s***t*)*p*(*.*) | 1287.748 | 19.7942 | 0.00002 | 27 | 0.00 | 1.281 |
| $\hat{\theta}$(*s***t*)*p*(*t*) | 1296.404 | 28.4499 | 0 | 46 | 0.01 | 1.363 |

The16 top-ranked models from the capture matrix with only individuals having sex determined (1360 individuals) are shown. Models include probability of survival ($\hat{\theta}$) and capture (*p*) with all combinations of sex (*s*) and time (*t*). Shown are Akaike’s information criterion corrected for small samples (AIC*_c_*) difference between the top-ranked model AIC*_c_* and the current model (δAIC*_c_*), AIC*_c_*weights (*w*AIC*_c_*) and the number of estimable parameters (*k*). From bootstrap goodness-of-fit tests the probability of a deviance less than or equal to the observed deviance from 100 bootstrap goodness-of-fit simulations of the model (*P*), and the quasi likelihood over (or under) dispersion factor ($\hat{c})$ area also presented.
