# Supplementary material for: Nautilus at Risk – Estimating Population Size and Demography of Nautilus pompilius
Source: PLoS One. 2011 Feb 10;6(2):e16716. doi: 10.1371/journal.pone.0016716 (PMC3037370; doi:10.1371/journal.pone.0016716)
Supplement: Table S3 — POPAN goodness-of-fit summaries for highest ranking population models. Two different matrices were analysed; one with all individuals (1553 total) present and one with only individuals having sex determined (1360 total) (sex parameter Y/N column). Models shown are the highest ranking models and include probability of survival () and capture (p) with combinations of sex (s) and time (t). Shown are Akaike's information criterion corrected for small samples (AICc), AICcweights (wAICc) and the number of estimable parameters (k). (DOCX) [file pone.0016716.s004.docx]

**Table S3. POPAN goodness-of-fit summaries for highest ranking population models.**

| Sex grouping | Model | AICc Weights | Num. Par |
| --- | --- | --- | --- |
| Y | $\hat{\theta}\left( t \right)p\left( t \right)\beta\left( . \right)N(.)$ | 0.99991 | 46 |
| Y | $\hat{\theta}\left( t \right)p\left( s*t \right)\beta\left( . \right)N(.)$ | 0.00008 | 68 |
| Y | $\hat{\theta}\left( s*t \right)p\left( s*t \right)\beta\left( . \right)N(.)$ | 0.00001 | 87 |
| Y | $\hat{\theta}\left( t \right)p\left( t \right)\beta\left( t \right)N(.)$ | 0 | 66 |
| N | $\hat{\theta}\left( t \right)p\left( t \right)\beta\left( t \right)N(.)$ | 0.9786 | 59 |
| N | $\hat{\theta}\left( t \right)p\left( t \right)\beta\left( . \right)N(.)$ | 0.0214 | 44 |
| N | $\hat{\theta}\left( t \right)p\left( . \right)\beta\left( . \right)N(.)$ | 0 | 23 |
| N | $\hat{\theta}\left( t \right)p\left( . \right)\beta\left( t \right)N(.)$ | 0 | 21 |

Two different matrices were analysed; one with all individuals (1553 total) present and one with only individuals having sex determined (1360 total) (sex parameter Y/N column). Models shown are the highest ranking models and include probability of survival ($\hat{\theta}$) and capture (*p*) with combinations of sex (*s*) and time (*t*). Shown are Akaike’s information criterion corrected for small samples (AIC*_c_*), AIC*_c_*weights (*w*AIC*_c_*) and the number of estimable parameters (*k*).
