# Supplementary material for: Nautilus at Risk – Estimating Population Size and Demography of Nautilus pompilius
Source: PLoS One. 2011 Feb 10;6(2):e16716. doi: 10.1371/journal.pone.0016716 (PMC3037370; doi:10.1371/journal.pone.0016716)
Supplement: Table S4 — Nautilus horizontal movement speeds from remote telemetry studies. Nautiluses tagged with ultrasonic transmitters were tracked for extended periods. Records of maximum speeds over short term (<6hrs) and longer term (>7 days) are shown for individual tagged animals (Nautilus ID) of known sex and shell diameter. Distance travelled and time taken is presented with the average speed calculated. (DOCX) [file pone.0016716.s005.docx]

**Table S4. *Nautilus* horizontal movement speeds from remote telemetry studies.**

| **Short term fast movements** | | | | | |
| --- | --- | --- | --- | --- | --- |
| *Nautilus* **ID** | **Sex** | **Shell diameter (mm)** | **Distance (km)** | **Time (hrs)** | **Speed (km/hr)** |
| 78 | M | 129 | 2.169 | 1:50 | 1.18 |
| 78 | M | 129 | 3.241 | 5:17 | 0.61 |
| 79 | M | 131 | 1.446 | 1:54 | 0.76 |
| 81/82 | M | 132 | 2.703 | 3:14 | 0.84 |
| **Long distance movements** | | | | | |
| *Nautilus* **ID** | **Sex** | **Shell diameter (mm)** | **Distance (km)** | **Time (days)** | **Speed (km/day)** |
| 78 | M | 129 | 29.3 | 52.3 | 0.56 |
| 79 | M | 131 | 10.06 | 7.95 | 1.27 |
| 79 | M | 131 | 12.48 | 12 | 1.04 |
| 80 | F | 118 | 5.502 | 16 | 0.34 |
| 81/82 | M | 132 | 10.06 | 9.1 | 1.11 |
| 81/82 | M | 132 | 24.19 | 28.8 | 0.84 |
| 83 | M | 130 | 11.34 | 30.4 | 0.37 |

*Nautiluses* tagged with ultrasonic transmitters were tracked for extended periods. Records of maximum speeds over short term (<6hrs) and longer term (>7 days) are shown for individual tagged animals (Nautilus ID) of known sex and shell diameter. Distance travelled and time taken is presented with the average speed calculated.
