# Supplementary material for: Nautilus at Risk – Estimating Population Size and Demography of Nautilus pompilius
Source: PLoS One. 2011 Feb 10;6(2):e16716. doi: 10.1371/journal.pone.0016716 (PMC3037370; doi:10.1371/journal.pone.0016716)
Supplement: Table S5 — Nautilus capture and recapture data from Osprey Reef sampling sites. Data for Nautilus captures and recaptures at all sampling sites on Osprey Reef. All recaptures were originally trapped at the Entrance site and recaptures at other sites demonstrate the movement of individuals around the entire perimeter of Osprey Reef (Fig. 2). (DOCX) [file pone.0016716.s006.docx]

**Table S5. *Nautilus* capture and recapture data from Osprey Reef sampling sites.**

| **Location** | **Distance from Entrance** | **# samples** | **# captures** | **# recaptures** | **% recaptures** |
| --- | --- | --- | --- | --- | --- |
|  | (nm) |  |  |  |  |
| **Entrance** | **0** | **268** | **1553** | **157** | **10.1** |
| False Entrance | 1 | 5 | 17 | 3 | 17.6 |
| Castles | 2.5 | 14 | 76 | 8 | 10.5 |
| North Horn | 5.3 | 3 | 9 | 3 | 33.3 |
| Pavona | 4.3 | 6 | 60 | 8 | 13.3 |
| Osprey east wall | 8.3 | 1 | 11 | 2 | 18.2 |
| Rapid Horn | 12 | 2 | 8 | 0 | 0.0 |
| SE corner | 18 | 4 | 26 | 2 | 7.7 |
| **TOTAL** |  | **303** | **1760** | **183** |  |

Data for *Nautilus* captures and recaptures at all sampling sites on Osprey Reef. All recaptures were originally trapped at the Entrance site and recaptures at other sites demonstrate the movement of individuals around the entire perimeter of Osprey Reef (Fig.2).
